# Structure of a xenon derivative of *Escherichia coli* copper amine oxidase: confirmation of the proposed oxygen-entry pathway

**DOI:** 10.1107/S1744309108036373

**Published:** 2008-11-28

**Authors:** Pascale Pirrat, Mark A. Smith, Arwen R. Pearson, Michael J. McPherson, Simon E. V. Phillips

**Affiliations:** a Astbury Centre for Structural Molecular Biology, University of Leeds, Leeds LS2 9JT, England

**Keywords:** copper amine oxidase, xenon derivatives, oxygen entry

## Abstract

The mechanism of molecular oxygen entry into the buried active site of the copper amine oxidase family has been investigated in several family members using biochemical, structural and *in silico* methods. These studies have revealed a structurally conserved β-sandwich which acts as a hydrophobic reservoir from which molecular oxygen can take several species-specific preferred pathways to the active site. *Escherichia coli* copper amine oxidase (ECAO) possesses an extra N-terminal domain that lies close to one entrance to the β-sandwich. In order to investigate whether the presence of this domain alters molecular oxygen entry in this enzyme, xenon was used as a molecular oxygen binding-site probe. The resulting 2.5 Å resolution X-ray crystal structure reveals xenon bound in similar positions to those observed in xenon-derivative crystal structures of other family members, suggesting that the N-terminal domain does not affect oxygen entry and that the *E. coli* enzyme takes up oxygen in a similar manner to the rest of the copper amine oxidase family.

## Abstract 2

The structure of a xenon derivative of *E. coli* copper amine oxidase confirms the pathway of oxygen entry to the buried active site proposed for this class of enzymes.

## 1. Introduction

**Figure 1:**
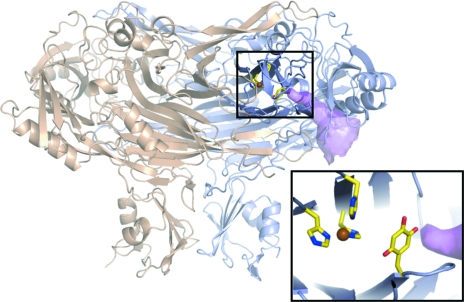
The structure of ECAO (PDB code 1dyu) showing the substrate-entrance channel (magenta surface) and active site (Murray *et al.*, 1999 ▶). The lower right panel is a more detailed view of the active site showing the TPQ, copper(II) ion, three coordinating histidine residues (sticks coloured by atom type) and the end of the substrate-entrance channel (magenta surface). The substrate-entrance channel was calculated using *CAVER* (Petrek *et al.*, 2006 ▶) and the image was prepared using *PyMOL* (DeLano, 2002 ▶).

**Figure 2:**
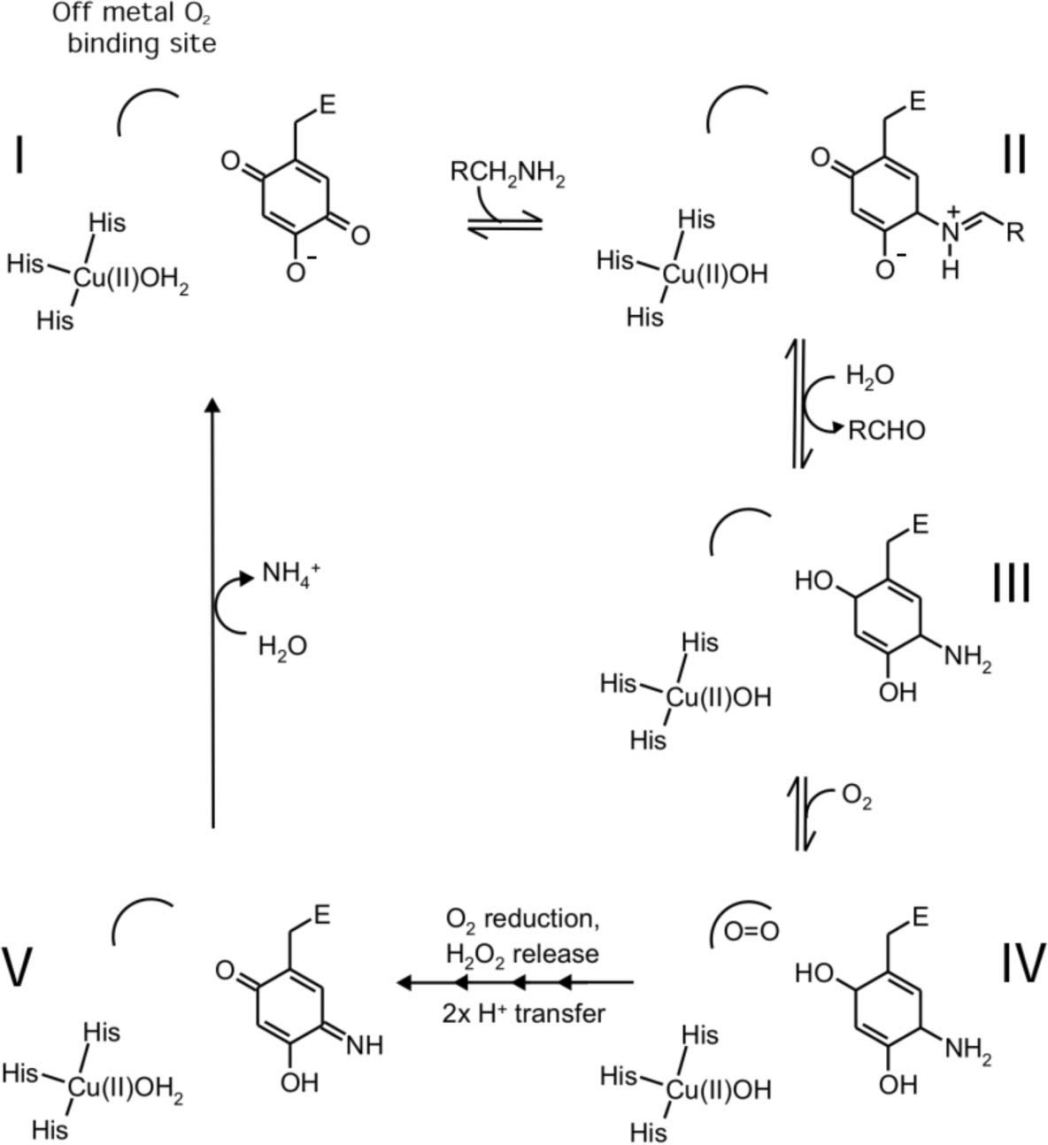
Schematic catalytic cycle of CuAOs identifying the key intermediates in the reaction pathway. Shown are the type-2 copper centre, the protein-derived cofactor TPQ and the off-metal O_2_-binding site located in the vicinity of a conserved active-site methionine (Met699 in ECAO). I is the resting state of the enzyme, intermediate II is the product Schiff base, intermediate III is the reduced TPQ or aminoquinol form, intermediate IV shows the proposed off-metal-bound dioxygen species prior to oxygen activation and reduction and intermediate V is the iminoquinone species which hydrolyses to release ammonia, completing the catalytic cycle.

Copper amine oxidases (CuAOs) catalyse the oxidation of a wide range of amine substrates to generate a product aldehyde, ammonia and hydrogen peroxide (Mure *et al.*, 2002 ▶). They are ubiquitous in nature and in humans are involved in diverse processes including cell adhesion and signalling (Salmi *et al.*, 1998 ▶; Elmore *et al.*, 2002 ▶; Yu *et al.*, 2003 ▶). In bacteria, they play a role in general nitrogen catabolism (Parrott *et al.*, 1987 ▶; Hacisalihoglu *et al.*, 1997 ▶). They contain a protein-derived cofactor that is generated autocatalytically by post-translation modification of a conserved endogenous tyrosine to generate 2,4,5-trihydroxylphenylalanine quinone (TPQ), requiring only the presence of copper and molecular oxygen (Cai & Klinman, 1994 ▶; Kim *et al.*, 2002 ▶). TPQ is situated in the core of the protein at the end of a well defined substrate-access channel next to a type II copper coordinated by three histidines (Fig. 1 ▶; Parsons *et al.*, 1995 ▶; Duff *et al.*, 2006 ▶; Wilce *et al.*, 1997 ▶; Lunelli *et al.*, 2005 ▶; Kumar *et al.*, 1996 ▶; Airenne *et al.*, 2005 ▶; Li *et al.*, 1998 ▶). The *Escherichia coli* enzyme (ECAO) shows a preference for small aromatic amine substrates such as phenethylamine and tyramine (Roh *et al.*, 1994 ▶). The catalytic reaction proceeds *via* two half-reactions; the aldehyde product is released at the end of the reductive half-reaction before reduction of molecular oxygen in the oxidative half-reaction (Fig. 2 ▶).

Extensive structural and biochemical analyses of CuAOs from prokaryotic and eukaryotic sources have revealed that molecular oxygen binds on the opposite side of TPQ to the amine substrate (Fig. 2 ▶). There has been considerable interest in dissecting the pathway by which oxygen reaches and binds in the active site. Structural and mutagenesis studies have shown that oxygen does not enter *via* the substrate channel and that other possibly specific oxygen-access pathways must be present (Wilce *et al.*, 1997 ▶; Li *et al.*, 1998 ▶; Johnson *et al.*, 2007 ▶; Wilmot *et al.*, 1999 ▶; Goto & Klinman, 2002 ▶).

**Figure 3:**
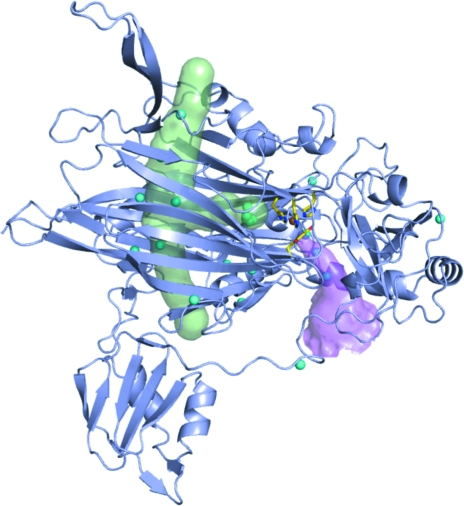
ECAO subunit showing the substrate-entry channel as a magenta surface and the putative oxygen-entry pathway as a green surface (generated by placing dummy atoms along the channel). The xenon sites observed in PPLO, HPAO, AGAO and PSAO are shown as cyan spheres. TPQ and copper ligands are shown as sticks coloured by element and copper is shown as a bronze sphere. This image was generated using *PyMOL* (DeLano, 2002 ▶).

Several recent crystallographic studies of oxygen entry have been carried out using xenon as a probe to identify possible molecular oxygen-binding sites that may be part of the putative oxygen-entry pathway (Lunelli *et al.*, 2005 ▶; Johnson *et al.*, 2007 ▶; Duff *et al.*, 2004 ▶). Xenon-complex crystal structures are now available for the CuAOs from *Hansenula polymorpha* (HPAO), *Pisum sativum* (PSAO), bovine serum (BSAO), *Pichia pastoris* (PPLO) and *Arthobacter globiformis* (AGAO) (Lunelli *et al.*, 2005 ▶; Johnson *et al.*, 2007 ▶; Duff *et al.*, 2004 ▶). These structures have revealed a consistent pattern of xenon binding that has implicated a major pathway for oxygen entry through a conserved β-sandwich (Fig. 3 ▶). Very recently, *in silico* experiments that calculated likely low-energy pathways for oxygen entry in HPAO, PSAO, AGAO and PPLO were carried out that supported the major pathway identified by the structural studies (Johnson *et al.*, 2007 ▶).

ECAO is distinct from Gram positive bacterial and yeast CuAOs in that it possesses an extra 9.3 kDa N-terminal domain, which gives ECAO a characteristic ‘mushroom stalk and cap’ shape (Parsons *et al.*, 1995 ▶). Mammalian CuAOs also possess an N-terminal domain that is thought to act as a membrane anchor (Smith *et al.*, 1998 ▶) but has not yet been visualized crystallographically (Airenne *et al.*, 2005 ▶). Bio­chemical and structural characterization of ECAO indicate that the binding of oxygen in the active site is similar to that of the other family members (Smith & McPherson, 2008 ▶); however, it is possible that the presence of the additional N-terminal domain, which is located at one end of the structurally conserved β-sandwich, could alter or provide an alternate pathway for oxygen entry. In order to investigate this possibility, we have determined the crystal structure of ECAO in complex with xenon at 2.5 Å resolution.

## 2. Experimental

### 2.1. ECAO expression, purification and crystallization

ECAO expression and purification was carried out as previously described (Murray *et al.*, 1999 ▶). ECAO was concentrated to 8 mg ml^−1^ and crystals were grown by vapour diffusion at 291 K. Large pink-coloured crystals grew over two weeks in 100 m*M* HEPES pH 7 and 1.2 *M* sodium citrate.

### 2.2. Xenon derivatization

ECAO crystals were harvested and transferred into a cryoprotectant solution comprising crystal-growth mother liquor containing 20% glycerol before being exposed to xenon for 8 min at 2.5 MPa in an Oxford Cryosystems Xcell. After derivatization, crystals were immediately flash-cooled in liquid nitrogen for X-ray data collection.

### 2.3. X-ray data collection and processing

A 2.5 Å diffraction data set was collected on beamline 14.1 at the SRS, Daresbury. Data collection was carried out at the highest beamline-accessible wavelength of 1.48 Å to maximize the anomalous signal from xenon (Xe *f*′′ = 6.9) and data were recorded using a Quantum4 ADSC CCD detector. The crystal was maintained at 100 K using a Cryostream (Oxford Cryosystems). 360° of data were collected with an oscillation angle of 1° and an exposure time of 30 s. The diffraction data were processed using *MOSFLM* and scaled using *SCALA* (Leslie, 1992 ▶; Evans, 1993 ▶; Table 1 ▶).

### 2.4. Refinement and model building

All refinement and model building was carried out using the *CCP*4 software suite and *Coot* (Collaborative Computational Project, Number 4, 1994 ▶; Emsley & Cowtan, 2004 ▶). Initial maps were calculated by direct Fourier transform using the structure of native ECAO (PDB code 1dyu; Murray *et al.*, 1999 ▶). An anomalous difference map was calculated and examined to locate possible xenon sites. 50 peaks were identified in the map with a σ level of 4 or above. Two of these (8.5 and 8.0 r.m.s.) were located at sites known to bind calcium in the native enzyme (Ca *f*′′ = 1.2 at 1.48 Å). Weaker peaks (3.6–4.6 r.m.s.) were located at the copper site and on S atoms, which is expected at this wavelength (Cu *f*′′ = 0.55 at 1.48 Å, S *f*′′ = 0.5 at 1.48 Å). The remaining peaks were assigned as xenon when they were higher than 4 r.m.s., did not overlap with copper, calcium or sulfur, corresponded to positive electron density in the *F*_o_ − *F*_c_ electron-density map and were located in mainly hydrophobic pockets. The occupancy of each xenon was modified such that after restrained refinement there was no further positive electron density in the *F*_o_ − *F*_c_ map and the *B* factors were similar to those of nearby atoms. The structure was further refined using iterative cycles of model building and restrained refinement to a final *R* factor of 16.1% and an *R*_free_ of 21.9% (Table 2 ▶). Structure validation was carried out using *SFCHECK* and *PRO­CHECK* (Laskowski *et al.*, 1993 ▶; Vaguine *et al.*, 1999 ▶).

## 3. Results and discussion

**Figure 4:**
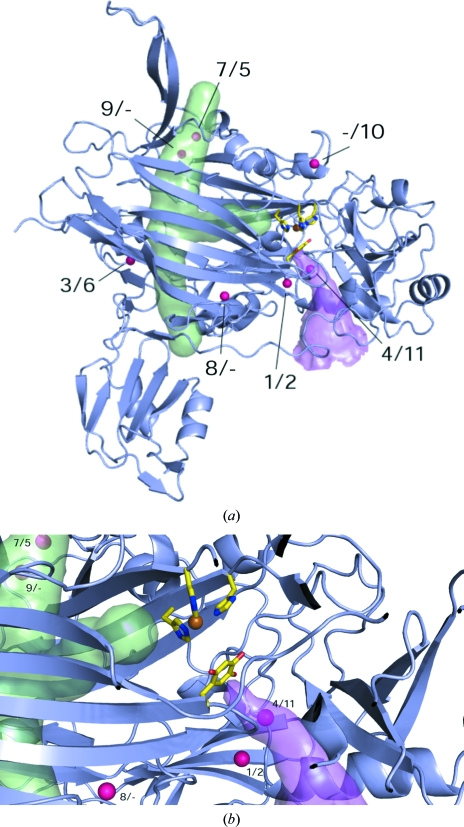
(*a*) ECAO monomer showing the substrate-entry channel as a magenta surface and the putative oxygen-entry pathway as a green surface (generated by placing dummy atoms along the channel). The Xe sites observed in ECAO are shown as magenta spheres. TPQ and copper ligands are shown as sticks coloured by element and copper is shown as a bronze sphere. (*b*) ECAO active site, coloured as before, showing xenons 1/2, 4/11, 7/5, 8/– and 9/–. This image was generated using *PyMOL* (DeLano, 2002 ▶).

After refinement, 11 xenon-binding sites were found in the structure (Fig. 4 ▶ *a*). Although the occupancy of all sites was low (0.1–0.45), this is consistent with the xenon occupancies observed in other CuAO xenon complexes (Lunelli *et al.*, 2005 ▶; Johnson *et al.*, 2007 ▶; Duff *et al.*, 2004 ▶). Xenons were numbered according to their occupancy (from high to low occupancy). The Xe atoms were bound in hydrophobic pockets located in either the amine substrate-entry channel or around the conserved β-sandwich oxygen-entry pathway (Table 3 ▶). Nearly identical sites were observed in both subunits of ECAO present in the asymmetric unit^1^.

Two Xe atoms are bound in the amine substrate-entry channel. Xenon 4/11 occupies a site between Tyr381 and Tyr387 that is equivalent to that previously observed in AGAO and HPAO. Tyr381 is proposed to act as a gate regulating substrate access to the TPQ and the aromatic rings of Tyr381 and Tyr387 allow stacking inter­actions with the aromatic moiety of aromatic amine substrates (Wilmot *et al.*, 1997 ▶, 2004 ▶). Xenon 1/2 is bound further from the active site but is still close to the amine-substrate channel (Fig. 4 ▶ *b*).

Two xenons, xenon 7/5 and xenon 9/– (only in monomer *A*), are bound inside the ‘top’ entrance of the β-sandwich, close to the xenon-binding sites observed in PPLO and PSAO (Duff *et al.*, 2004 ▶; Figs. 4 ▶ *a* and 4 ▶ *b*). We did not observe any xenon-binding sites inside the lower half of the β-sandwich near the ‘bottom’ entrance to the putative oxygen-entry pathway. However, two xenon-binding sites (3/6 and 8/–) flank the lower half of the β-sandwich, binding on its external face (Fig. 4 ▶ *a*). This is consistent with the other copper amine oxidase–xenon complexes, in which a xenon-binding site within the bottom half of the β-sandwich channel has only been reported for AGAO (Fig. 3 ▶). The lack of xenon-binding sites in this region in PPLO, PSAO and HPAO suggests that xenon binding is not as favourable in this region of the β-sandwich regardless of the presence or absence of an N-­terminal domain. A final xenon, xenon –/10, is bound at the edge of ECAO in a hydrophobic pocket and is not close to any previously observed xenon position.

The xenon-complex ECAO structure reported here shows that molecular oxygen is likely to access the ECAO active site by very similar pathways to those proposed by experimental and *in silico* studies in other CuAOs (Lunelli *et al.*, 2005 ▶; Johnson *et al.*, 2007 ▶; Duff *et al.*, 2004 ▶). No xenon-binding sites are observed in the ECAO N-­terminal domain itself nor does the presence of this domain appear to affect xenon binding in the lower half of the β-sandwich when compared with other CuAOs for which xenon complexes have been determined. These observations indicate that the N-terminal domain does not play a role in oxygen entry during ECAO catalysis, which is consistent with the lack of conservation of the N-terminal domain across the CuAOs.

## Supplementary Material

PDB reference: *E. coli* copper amine oxidase, xenon derivative, 2w0q, r2w0qsf

## Figures and Tables

**Table 1:** Data collection and processing Values in parentheses are for the highest resolution shell.

Wavelength (Å)	1.48
Temperature (K)	100
Unit-cell parameters (Å)	*a* = 166.9, *b* = 134.6, *c* = 80.3
Space group	*P*2_1_2_1_2_1_
Resolution (Å)	50.0–2.5 (2.55–2.50)
*R*_merge_† (%)	12.3 (44.8)
Completeness (%)	98.6 (94.9)
Redundancy	10.1 (7.4)
Mean *I*/σ(*I*)	23.6 (4.4)

† *R*_merge_ = 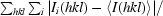 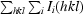, where *I_i_*(*hkl*) is the observed intensity and 〈*I*(*hkl*)〉 is the average intensity for multiple measurements.

**Table 2:** Structure refinement and validation Values in parentheses are for the highest resolution shell.

*R*_work_†	16.1 (21.9)
*R*_free_‡	21.9 (30.1)
Total No. of reflections	64552
No. of reflections in the *R*_work_ set	61345 (3900)
No. of reflections in the *R*_free_ set	3207 (220)
No. of non-H atoms	11947
No. of protein atoms	11348
No. of ligand atoms	17
No. of solvent atoms	582
R.m.s.d. from ideality	
Bonds (Å)	0.017
Angles (°)	1.7
Average *B* factors (Å^2^)	
Main chain	39.6
Side chain	41.5
Ligands	49.4
Solvent	44.7
Ramachandran plot	
Allowed regions (%)	99.8
Disallowed regions (%)	0.2
PDB code	2w0q

† *R* factor = 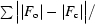 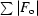, where |*F*_o_| is the observed structure-factor amplitude and |*F*_o_| is the calculated structure-factor amplitude.

‡ *R*_free_ is the *R* factor based on 5% of the data excluded from refinement.

**Table 3:** Xenon sites

Xenon site	Peak height	Occupancy/*B* factor (Å^2^)	Surrounding residues
1	13	0.45/58	Subunit *A* Leu189, Phe192, Phe387, Leu392
2	9.9	0.30/48	Subunit *B* Leu189, Phe192, Tyr387, Leu392
3	9.9	0.30/39	Subunit *A* His94, Ala140, Met322, Thr344
4	6.5	0.35/57	Subunit *A* Phe192, Pro224, Leu225, Tyr381, Tyr387
5	5.8	0.15/48	Subunit *B* Leu543, Ile570, Gln578, Leu638
6	5.7	0.3/57	Subunit *B* His94, Ala140, Met322, Thr344
7	5.6	0.2/46	Subunit *A* Leu543, Ile570, Gln578, Leu638
8	4.9	0.15/27	Subunit *A* Ile396, Ile460
9	4.5	0.1/37	Subunit *A* Leu543, Leu588, Ile605, Leu638, Val681
10	4.5	0.15/47	Subunit *B* Pro458, Met563, Gln620
11	4.2	0.2/58	Subunit *B* Phe192, Pro224, Leu225, Tyr381, Tyr387

## Footnotes

1 As ECAO is a dimer in the asymmetric unit some xenon sites are observed in equivalent positions in both monomers, while others are observed in only one of the subunits. In the following discussion of individual xenon sites, those observed in both monomers are identified as ‘xenon *A*/*B*’, where *A* is the xenon number in monomer *A* and *B* that in monomer *B*. When a xenon is observed in only a single subunit, this is indicated as either ‘xenon *A*/–’ or ‘xenon –/*B*’.
